# Prevalence of Positive Mental Health and Associated Factors Among Postpartum Women in Canada: Findings from a National Cross-Sectional Survey

**DOI:** 10.1007/s10995-020-02920-8

**Published:** 2020-04-22

**Authors:** Mélanie Varin, Elia Palladino, Heather M. Orpana, Suzy L. Wong, Mihaela Gheorghe, Tanya Lary, Melissa M. Baker

**Affiliations:** 1 Indigenous Services Canada, Ottawa, Canada; 2 Public Health Agency of Canada, 785 Carling Avenue, Ottawa, Canada; 3 School of Epidemiology and Public Health, University of Ottawa, Ottawa, Canada; 4 Royal Institute of Mental Health Research, Ottawa, Canada; 5 Women and Gender Equality Canada, Gatineau, QC Canada

**Keywords:** Maternal health, Positive mental health, Physical health, Life satisfaction, Sense of community belonging, Public health

## Abstract

**Objectives:**

The objectives of this study were to compare the prevalence of three positive mental health (PMH) outcomes (self-rated mental health, life satisfaction, sense of community belonging) in postpartum women to the general population, and to examine the relationship between protective factors and the three PMH outcomes among postpartum women.

**Methods:**

The national cross-sectional Survey on Maternal Health (n = 6558) was analyzed. Analyses were weighted and 95% confidence intervals were calculated. Three adjusted logistic regression models were generated. To compare this sample to the general population of women, estimates from the Canadian Community Health Survey—Annual Component (2018) were used.

**Results:**

Compared to the general population of women, a larger proportion of postpartum women reported a strong sense of community belonging. The odds of postpartum women with high self-rated physical health having high self-rated mental health were approximately seven times greater (aOR 6.9, 95% confidence interval [CI] 5.9, 8.1) than postpartum women with lower self-rated physical health. The absence of symptoms of postpartum depression (PPD) or generalized anxiety disorder (GAD) and high self-rated physical health were significantly associated with all three PMH outcomes. Frequent availability of maternal support was associated with greater odds of high life satisfaction (aOR 1.6, 95% CI 1.4, 1.9) and sense of community belonging (aOR 1.4, 95% CI 1.2, 1.6).

**Conclusions:**

Our study demonstrated that availability of maternal support, self-rated physical health and absence of symptoms of PPD or GAD were associated with PMH among postpartum women. As physical health had the strongest association with mental health, we encourage further examination of this relationship.

**Electronic supplementary material:**

The online version of this article (10.1007/s10995-020-02920-8) contains supplementary material, which is available to authorized users.

Our study addresses a current gap in literature by focusing on *positive* rather than *negative* mental health among postpartum women. We provided nationally representative prevalence estimates for three positive mental health outcomes in a sample of postpartum women and compared them to the general population. Our findings indicate that postpartum women had a higher prevalence of strong sense of community belonging. We also found that high self-rated physical health, having no symptoms of postpartum depression or generalized anxiety disorder, having maternal support available all or most of the time, and being married or in a common-law relationship were positively and significantly associated with one or more positive mental health outcome(s).

## Introduction

Mental health is a public health priority, as it is an integral part of the overall health of the population. Across the lifespan, mental health is dynamic and may be influenced by life events such as pregnancy and childbirth (Brummelte and Galea 2016). Physiological (Brummelte and Galea 2016), psychological (O'Hara 2009; Robertson et al. 2004) and other changes that occur during pregnancy and the postpartum period may contribute to changes to a woman’s mental health status, including an increased risk for symptoms of mood and anxiety disorders (Brummelte and Galea 2016; O'Hara 2009; Robertson et al. 2004). Global prevalence of postpartum depression and anxiety among women has been estimated to range between 10 to 20% (Brummelte and Galea 2016; Schmied et al. 2013; Yelland et al. 2010) and 8 to 13% (Schmied et al. 2013; Yelland et al. 2010), respectively. These estimates are particularly important as several studies have established a relationship between postpartum depression and negative outcomes among women and children, including premature delivery (Dayan et al. 2006), breastfeeding difficulties (Dennis and McQueen 2009), behavioural problems, poor executive functioning, and adverse health-related quality of life (Park et al. 2018). While these studies provide a strong evidence base, they are limited in that they solely focused on mental illness.

The Public Health Agency of Canada (PHAC) defines positive mental health (PMH) as “the capacity of each and all of us to feel, think, and act in ways that enhance our ability to enjoy life and deal with the challenges we face” (Public Health Agency of Canada 2014). Promoting PMH can strengthen individuals and communities, increase overall physical health, and decrease the risk factors for poor mental health (Public Health Agency of Canada 2014). Research focusing on PMH among postpartum women is scarce, but a greater understanding of positive mental functioning in this period may be important for both women and families. To our knowledge, this is the first national study examining PMH outcomes in postpartum women. We aimed to (1) compare three PMH outcomes in postpartum women to the general population of women in Canada, and (2) examine the relationship between socio-demographic factors, social support, physical health, and PMH outcomes among postpartum women.

## Methods

### Sample of Postpartum Women

We used data from the 2018 to 2019 Survey on Maternal Health, conducted by Statistics Canada in collaboration with PHAC. The survey used the Canada Child Benefit (tax-free monthly payment administered to eligible families with children under 18) as the sampling frame to identify women in Canada who had given birth between January 1 and June 30, 2018. It is estimated that the Canada Child Benefit sampling frame is representative of 96% of the population of children (Statistics Canada 2018). Women living in the three territories, Indigenous Reserves, and institutions were excluded from the survey’s coverage. The survey was administered from November 29, 2018 to February 5, 2019 and the total survey sample consisted of 13,000 dwellings in 10 provinces. The response rate was 54.7%, which resulted in a sample size of 7085 women. Data were collected through an electronic questionnaire on the Statistics Canada website, accessed using a unique code that was provided to women who were invited to participate. Women who did not complete the electronic questionnaire were subsequently contacted by Statistics Canada interviewers to complete the survey by computer-assisted telephone interview. Of the 7085 survey respondents, 6558 agreed to share their data with PHAC and were therefore included in this study. Data were collected under the authority of the Statistics Act (Government of Canada 1985), and confidentiality rules were applied to prevent disclosure of any confidential or identifiable information. Participation in this survey was voluntary and respondents gave their informed consent to take part in the study. PHAC signed a data sharing agreement with Statistics Canada to get access to information provided in the survey.

### National Sample

The Canadian Community Health Survey—Annual Component 2018 is a national cross-sectional survey of the population in Canada aged 12 years and older conducted by Statistics Canada. The Canada Child Benefit frame and Area frame were used to identify the survey sample. Women living on reserves or other Aboriginal settlements, full-time members of the Canadian Armed Forces and individuals in institutions were excluded from the survey coverage. These exclusions represent less than 3% of the population aged 12 years and over. To produce a sample representative of the general population that could be compared to women who participated in the Survey on Maternal Health, we restricted the sample to women between the ages of 17–55 who had not given birth in the last five years. In total, 10,632 women were included in the final sample size. This survey was only used to address our first objective.

This study was based on aggregated, de-identified secondary data shared with PHAC under the authority of the Federal Statistics Act (Government of Canada 1985). Therefore, research ethics board approval was not required. The analyses are available from the corresponding author on reasonable request. We used the Reporting of studies conducted using observational routinely-collected health data (RECORD) to report the results from this study (Benchimol et al. 2015).

## Measures

### PMH Outcomes

Both the Survey on Maternal Health and the Canadian Community Health Survey included three PMH-related measures: self-rated mental health, life satisfaction, and sense of community belonging. Self-rated mental health was measured with the question: “In general, how would you rate the following: your mental health?” Response options included “excellent, very good, good, fair, and poor”. High mental health was defined as respondents who rated their mental health as “excellent” or “very good”. Life satisfaction was assessed with the question: “Using a scale of 0 to 10, where 0 means “Very dissatisfied” and 10 means “Very satisfied”, how do you feel about your life as a whole right now?” High life satisfaction was defined as respondents who reported a score of eight or more on the scale. Sense of community belonging was measured with the question: “How would you describe your sense of belonging to your local community?” Response options included “very strong, somewhat strong, somewhat weak or very weak”. Strong sense of community belonging was defined as respondents who reported their sense of community belonging as “very strong” or “strong”. Not stated responses were considered as missing.

### Socio-Demographic

Age (continuous), educational attainment and marital status (dichotomized into married/common law and never married/separated/divorced or widowed) were included as socio-demographic measures. Educational attainment was categorized as less than high school (less than high school diploma or its equivalent), high school graduate (high school diploma or a high school equivalency certificate), and post-secondary graduate (trades certificate or diploma, college/CEGEP/other non-university, university certificate or diploma below the bachelor’s level, bachelor’s degree, university certificate, diploma, degree above the BA level). Not stated responses were deemed as missing. Additional socio-demographic measures were not available from the Survey on Maternal Health.

### Physical Health, Mental Health, and Maternal Support

Self-rated physical health was assessed with the question: “In general, how would you rate the following: Your physical health?” Response options included “excellent, very good, good, fair, and poor”. For this study, high physical health was defined as respondents who rated their physical health as “excellent” or “very good”. Symptoms consistent with GAD or PPD were assessed with derived variables provided by Statistics Canada. Women who had a score of three or more on the GAD-2 screening tool were considered to have symptoms consistent with GAD (Kroenke et al. 2007; Nath et al. 2018). Women who scored 7 or higher on the 5-item version of the Edinburgh Postnatal Depression Scale one week before completing the survey were considered to have a moderate level of symptoms of PPD (Eberhard-Gran et al. 2007). We will refer to these variables as symptoms of GAD and symptoms of PPD throughout the rest of the article. Moreover, maternal support was measured with three questions: (1) “During your pregnancy with [your child], did you attend pregnancy support programs? For example, programs where you learned about having a healthy pregnancy or caring for your baby”: “Yes” or “No” (*prenatal classes on childbirth and online support groups were excluded*), (2) “Since the birth of your baby, did you attend any parenting support programs? For example, programs where you learn about caring for your child, breastfeeding support, parenting skills and infant or child development”: “Yes” or “No” (*online support groups were excluded*), (3) “Since the birth of your baby, when you needed other support, how often was it available? Would you say”: “none of the time”, “a little of the time”, “some of the time”, “most of the time”, “all of the time”. Companionship, assistance, online support groups and other types of support needed were included in this question. These questions were included as three separate variables in the analyses. Not stated responses to any of the questions above were deemed as missing.

### Statistical Analysis

Descriptive statistics including weighted proportions and 95% confidence intervals (CI) were calculated for all the women in the Survey on Maternal Health sample. To compare the PMH outcomes, we calculated the proportion of high self-rated mental health, high life satisfaction, and strong sense of community belonging for both the sample of postpartum women and the general population. All estimates were weighted and variance was estimated using bootstrap weights provided by Statistics Canada. Significant differences between prevalence estimates were determined through overlapping 95% CIs.

Three adjusted logistic regression models were used to examine the association between each of the three PMH outcomes, and socio-demographic factors, maternal support, symptoms of mental illness, and self-rated physical health. Respondents who had missing data on any of these variables were excluded from these regression analyses. Considering the complex survey design, variance for the weighted estimates and models was calculated using the bootstrap approach (with 1000 replicates). Survey and bootstrap weights were provided by Statistics Canada. All statistical analyses were executed using SAS Enterprise Guide version 7.1 (SAS Institute Inc., Cary, NC, USA).

## Results

Respondents’ socio-demographic characteristics are captured in Table 1. The mean age of women in our sample of postpartum women was 31.8. Most women reported having graduated from a post-secondary education (77.3%) and being married or in a common-law relationship (90.5%). The majority of postpartum women had no symptoms of GAD (86.2%), no symptoms of PPD (82.1%), did not attend a pregnancy support (77.0%) or parent support program (69.4%), and reported that maternal support was available all or most of the time (61.1%).

**Table 1:** Descriptive characteristics of study population and of the general population sample

	% of all postpartum women^a^ (95% CI)	% of women general population^b^ (95% CI)
*Age—(mean)*	31.8 (31.6, 31.9)	37.0 (36.8, 37.3)
*Age category*	*n* = 6526	*n* = 10,632
17–24 years	7.8 (7.0, 8.6)	20.4 (19.1, 21.6)
25–29 years	24.5 (23.2, 25.7)	12.5 (11.4, 13.5)
30–34 years	38.5 (37.1, 39.9)	10.3 (9.4, 11.3)
35–39 years	23.2 (22.0, 24.5)	9.7 (8.9, 10.5)
40–55 years	6.0 (5.3, 6.8)	47.1 (46.0, 48.3)
*Highest level of education respondent*	*n* = 6547	*n* = 10,632
Less than high school	6.2 (5.4, 6.9)	6.4 (5.7, 7.0)
High school graduate	16.6 (15.5, 17.7)	24.1 (22.7, 25.5)
Post-secondary graduate	77.3 (76.1, 78.5)	68.3 (66.8, 69.9)
*Marital status*	*n* = 6553	*n* = 10,619
Married/common law	90.5 (89.6, 91.5)	53.8 (52.3, 55.4)
Never married/separated/divorced/widowed	9.5 (8.5, 10.4)	46.2 (44.6, 47.7)
*Self-rated mental health*	*n* = 6529	*n* = 10,623
Excellent/very good	59.1 (57.6, 60.6)	61.5 (60.0, 63.1)
Good/fair/poor	40.9 (39.4, 42.4)	38.5 (36.9, 40.0)
*Life satisfaction*	*n* = 6549	*n* = 10,615
High life satisfaction	69.9 (68.6, 71.3)	69.7 (68.2, 71.1)
Low life satisfaction	30.1 (28.7, 31.4)	30.3 (28.9, 31.8)
*Sense of community belonging*	*n* = 6529	*n* = 10,552
Very strong/strong	74.2 (72.9, 75.5)	65.8 (64.2, 67.3)
Very weak/weak	25.8 (24.5, 27.1)	34.2 (32.7, 35.8)
*Self-rated physical health*	*n* = 6552	
Excellent/very good	59.1 (57.7, 60.5)	
Good/fair/poor	40.9 (39.5, 42.3)	
*Symptoms of generalized anxiety disorder*	*n* = 6541	
Yes	13.8 (12.8, 14.9)	
No	86.2 (85.1, 87.2)	
*Symptoms of postpartum depression*	*n* = 6503	
Yes	17.9 (16.8, 19.0)	
No	82.1 (81.0, 83.3)	
*Attended pregnancy support program*	*n* = 6548	
Yes	23.0 (21.8, 24.2)	
No	77.0 (75.8, 78.2)	
*Attended parent support program*	*n* = 6554	
Yes	30.6 (29.3, 31.9)	
No	69.4 (68.1, 70.7)	
*Availability of maternal support*	*n* = 6495	
All/most of the time	61.1 (59.8, 62.5)	
Some/little/none of the time	38.9 (37.5, 40.2)	

No estimates are provided because these variables were only asked of postpartum women (Survey on Maternal Health)

^a^All estimates from this column are from the Survey on Maternal Health

^b^All estimates from this column are from the Canadian Community Health Survey—Annual Component 2018

Prevalence estimates for the three PMH outcomes can be found in Table 1. Compared to the general population of women, postpartum women in this study had a higher prevalence of strong sense of community belonging (74.2% vs. 65.8%). Since CIs did not overlap, these differences were significant. While the prevalence estimate for high life satisfaction was slightly greater among postpartum women and the prevalence estimate for high self-rated mental health was slightly greater for women in the national sample, overlapping CIs indicate that these slight differences are likely insignificant.

Table 2 presents the results from three adjusted logistic regression models. High self-rated physical health was significantly related with high self-rated mental health (aOR 6.91, 95% CI 5.90, 8.10), high life satisfaction (aOR 1.60, 95% CI 1.33, 1.91) and strong sense of community belonging (aOR 1.23, 95% CI 1.04, 1.45). Women with no symptoms of PPD or no symptoms of GAD had greater odds (aORs between 1.37 and 3.91) of high self-rated mental health, high life satisfaction and strong sense of community belonging compared to women with symptoms of PPD or GAD. The odds of high life satisfaction and strong sense of community belonging were 62% (aOR 1.62, 95% CI 1.37, 1.91) and 37% (aOR 1.37, 95% CI 1.17, 1.60) greater for women with support available all or most of the time compared to women with support available some, little or none of the time. Marital status was only significantly associated with one of the three PMH outcomes. The odds of high life satisfaction for women who were married or in a common law relationship were 85% greater (aOR 1.85, 95% CI 1.37, 2.50) than women who were never married, separated, divorced or widowed. In contrast, women who reported not attending a parent support program had odds of high self-rated mental health that were 23% greater (aOR 1.23, 95% CI 1.02, 1.47) than women who reported attending a parent support program. All three PMH outcomes were statistically significantly associated with each other.

**Table 2:** Logistic regression models of relationships between socio-demographic variables, self-rated physical health, availability of maternal support, symptoms of depression and anxiety, support programs, and positive mental health outcomes among postpartum women

Variable	aOR self-rated mental health, 95% CI	aOR life satisfaction, 95% CI	aOR sense of community belonging, 95% CI
*Age*	1.01 (0.99, 1.02)	0.96 (0.95, 0.98)	1.01 (0.99, 1.02)
*Highest level of education respondent*			
Less than high school	1.00	1.00	1.00
High school graduate	0.96 (0.62, 1.49)	0.79 (0.53, 1.18)	0.87 (0.59, 1.27)
Post-secondary graduate	0.84 (0.56, 1.25)	0.84 (0.59, 1.20)	0.77 (0.54, 1.11)
*Marital status*			
Married/common law	1.29 (0.93, 1.79)	1.85 (1.37, 2.50)	1.03 (0.78, 1.36)
Never married/separated/divorced or widowed	1.00	1.00	1.00
*Self-rated mental health*			
Excellent/very good		2.96 (2.46, 3.58)	1.58 (1.32, 1.90)
Good/fair/poor		1.00	1.00
*Life satisfaction*			
High life satisfaction	2.95 (2.44, 3.55)		2.50 (2.10, 2.96)
Low life satisfaction	1.00		1.00
*Sense of community belonging*			
Strong or very strong	1.60 (1.33, 1.92)	2.51 (2.11, 2.98)	
Low or very low	1.00	1.00	
*Self-rated physical health*			
Excellent/very good	6.91 (5.90, 8.10)	1.60 (1.33, 1.91)	1.23 (1.04, 1.45)
Good/fair/poor	1.00	1.00	1.00
*Symptoms of generalized anxiety disorder*			
Yes	1.00	1.00	1.00
No	2.29 (1.77, 2.97)	1.70 (1.35, 2.14)	1.38 (1.12, 1.70)
*Symptoms of postpartum depression*			
Yes	1.00	1.00	1.00
No	3.91 (3.05, 5.00)	3.58 (2.87, 4.46)	1.37 (1.10, 1.70)
*Attended pregnancy support program*			
Yes	1.02 (0.85, 1.24)	0.95 (0.78, 1.15)	1.08 (0.90, 1.29)
No	1.00	1.00	1.00
*Attended parent support program*			
Yes	1.00	1.00	1.00
No	1.23 (1.02, 1.47)	1.10 (0.92, 1.31)	0.95 (0.81, 1.12)
*Availability of maternal support*			
All/most of the time	1.01 (0.85, 1.19)	1.62 (1.37, 1.91)	1.37 (1.17, 1.60)
Some/little/none of the time	1.00	1.00	1.00

Logistic models adjusted for age, educational attainment, marital status, self-rated mental health, life satisfaction, sense of community belonging, self-rated physical health, symptoms of GAD, symptoms of PPD, pregnancy support program attendance, parent support program attendance, and availability of maternal support

## Discussion

Recently, the World Health Organization identified maternal mental health as a growing international public health challenge (World Health Organization 2019). The majority of the recent literature on postpartum mental health tends to focus on poor mental health, mental health disorders or illnesses, such as depression and anxiety. To our knowledge, this study is the first to estimate the prevalence of three PMH outcomes in postpartum women in Canada and to examine its relationship with socio-demographic factors, availability of maternal support, support programs and physical health.

The first objective of this study was to report on the prevalence of three PMH outcomes among postpartum women and to compare these estimates to a nationally representative sample. Our findings indicate that a larger proportion of women in the postpartum period reported a strong sense of community belonging compared to women in the general population, and that based on the CIs this difference was significant. A possible explanation for this result is that women who have recently given birth are members of social groups, such as mother groups, which provide them with a sense of belonging and social connectedness (Seymour-Smith et al. 2017). In fact, based on the Social Identity Model of Identity Change theory (Iyer et al. 2009), women who are able to maintain existing group memberships (for example with their friends and family), and who are able to build new group memberships (for example with a mother’s group) should have better well-being (Seymour-Smith et al. 2017). Future age-matched case–control studies exploring this finding are needed to get a better understanding of this statistically significant difference.

Our findings that the three PMH outcomes were strongly related with one another are in accordance with current evidence (Lombardo et al. 2018). These relationships are also supported with a conceptual view of PMH consistent of high levels of each of emotional, psychological and social well-being (Keyes 2005; Orpana et al. 2016). Our study included one general measure of PMH (self-rated mental health), one of emotional well-being (life satisfaction) and one of social well-being (sense of community belonging), all of which were positively associated with each other in the logistic regression models. Further research should examine whether these measures are of a high order concept through second-order confirmatory factor analysis.

Of the associations between the covariates and the three PMH outcomes, the strongest association was found between self-rated physical health and self-rated mental health. Our results are consistent (Fleishman and Zuvekas 2007) with correlation results that found a stronger correlation between self-rated health with self-rated mental health compared to any other mental health measure (Fleishman and Zuvekas 2007). The authors speculated that this positive correlation may be due to the broader concept of self-rated health including an evaluation of mental health, resulting in some overlap in the measures (Fleishman and Zuvekas 2007). Additionally, physical and mental health share 20% of variance in common (Hays 1990), which supports the assumption of overlap between both health measures (Fleishman and Zuvekas 2007). Certainly, the relationship between physical health problems, such as tiredness, back pain, breast problems, painful perineum, and urinary incontinence, have been linked with postpartum depression (Woolhouse et al. 2014). Our study is unique as it also demonstrates a relationship between self-rated physical health and greater odds of positive, instead of negative, mental health outcomes.

We also found that women with no symptoms of PPD or GAD had greater odds of high self-rated mental health, high life satisfaction and strong sense of community belonging compared to women with symptoms. Our findings are supported by the dual continuum literature stating that mental health and mental illness are two separate but complimentary constructs that are related with one another (Headey 1993; Keyes et al. 2010). Based on this theory, mental health is more than the absence of mental illness (Gilmour 2014). Findings from a 2014 statistical report demonstrate (Gilmour 2014) that prevalence of mental illness was lower for Canadians with higher mental health (flourishing), whereas the prevalence of mental illness was higher for Canadians with lower mental health (languishing) (Gilmour 2014). Although we measured mental health differently, we also found an inverse relationship between mental health and mental illness, in that women with no symptoms of GAD or PPD had greater odds of high self-rated mental health, high life satisfaction and strong sense of community belonging.

We found a significant relationship between availability of maternal support with high life satisfaction and strong sense of community belonging, but not with high self-rated mental health. Although we could not find published evidence on maternal support and *positive* mental health during the postpartum period, existing evidence suggests an association between maternal support received in the postpartum period and *negative* mental health (Kim et al. 2014; Li et al. 2017; Schwab-Reese et al. 2017). Two epidemiological studies found that women who received little support had increased odds of postpartum depression (Kim et al. 2014; Li et al. 2017), and one epidemiological study found that women with higher support had a reduced risk of anxiety and depression (Schwab-Reese et al. 2017). Our study contributes to the literature by showing the benefits of frequent maternal support on life satisfaction and sense of community belonging for women in the postpartum period.

Conversely, we found that women who did not attend a parent support program had greater odds of high self-rated mental health compared to women who did. A potential explanation for this counterintuitive finding is that women who seek parenting programs might have unmeasured characteristics, such as previous diagnosis of depression/anxiety, stressful pregnancy and whether they have been pregnant before, which could confound the relationship. Furthermore, questions on pregnancy and parenting support programs excluded online support groups, which is particularly noteworthy since online support groups are used by women for information gathering and social connections during the postpartum period (Baker and Yang 2018; McDaniel et al. 2012; Teaford et al. 2019). Some evidence suggests that online support groups can impact decision-making (Lagan et al. 2011), well-being and mental health (McDaniel et al. 2012) for postpartum women. Since online support was not captured in our pregnancy and parenting support program measures, prudence is advised when interpreting our support program findings.

Finally, we found that the odds of high life satisfaction were superior for women who were in a married or common law relationship compared to women who were never married/separated/divorced or widowed. This finding complements existing literature, which shows that being unwed was a risk factor for serious psychological distress (Glasheen et al. 2015) among postpartum women. Since we adjusted for symptoms consistent with postpartum depression, caution is warranted when comparing our results to this evidence.

### Limitations and Strengths

While our findings are novel, this study is not without limitations. Due to the cross-sectional nature of the data, temporality cannot be established between the covariates and PMH. Since all of our data are based on self-report our results might be subject to shared methods variance and social desirability bias. However, it should be noted that all PMH outcomes are self-report as it is one’s subjective interpretation. Moreover, since the survey was conducted online, respondents might have felt more comfortable disclosing information on sensitive topics and the social desirability bias effect might have been diminished. Because of limited content, we were unable to adjust for additional factors such as previous pregnancy, income, immigrant status, obstetric complications, sleep, and parenthood stress, which could result in residual confounding. Since this survey was administered during the winter, our results might be subjected to seasonality effects. Furthermore, some overlap between the three PMH variables may exist as they are interrelated. In addition, only women who applied for the Canada Child Benefit (CCB) could participate in this survey, therefore women who did not apply for the CCB, or who experienced a stillbirth or a death of a child shortly after birth were excluded from the survey. Moreover, due to the survey exclusion criteria, these results are not representative of women living in the territories or on Indigenous reserves. Lastly, since our sample of women from the general population was not matched by age to our sample of postpartum women, caution is warranted when interpreting our comparison findings. Strengths of this study include the relatively large sample size, and relatively complete sampling frame, and the ability to compare the prevalence of three PMH outcomes to the general population. As a result of survey weighting, findings are generalizable to postpartum women in Canada living in the 10 provinces. Finally, we addressed a current gap in the literature on PMH among postpartum women.

## Conclusion

In conclusion, our study was the first to estimate the prevalence of three PMH outcomes in a nationally representative sample of postpartum women and compare to women from the general population. We found that the prevalence of strong sense of community belonging was higher in the sample of postpartum women compared to women in the general population. Future age-matched case–control analyses examining these three outcomes for women in the same sample is encouraged. In addition, our findings indicate that high self-rated physical health, having no symptoms of PPD, and no symptoms of GAD were significantly associated with all three PMH outcomes, whereas availability of maternal support was significantly related to two PMH outcomes, and being married or in a common law relationship was significantly associated with high life satisfaction only. Our findings also show that self-rated mental health, life satisfaction, and sense of community belonging were significantly related with each other. As our findings highlighted self-rated physical health as having the strongest association with self-rated mental health, further exploration of the relationship is encouraged. Future studies should examine these associations longitudinally throughout the antenatal, pregnancy and postpartum stage, which would provide insight on how PMH outcomes and associated determinants vary during this period.

## Electronic supplementary material

Below is the link to the electronic supplementary material.

Supplementary file1 (DOCX 20 kb)

## Abbreviations

PHAC: Public Health Agency of Canada
PMH: Positive mental health
GAD: Generalized anxiety disorder
PPD: Postpartum depression
